# Free and Modified Mycotoxins in Organic and Conventional Oats (*Avena sativa* L.) Grown in Scotland

**DOI:** 10.3390/toxins15040247

**Published:** 2023-03-28

**Authors:** Noshin Daud, Valerie Currie, Gary Duncan, Joao A. N. Filipe, Tomoya Yoshinari, Gary Stoddart, Deborah Roberts, Silvia W. Gratz

**Affiliations:** 1 Rowett Institute, University of Aberdeen, Foresterhill Health Campus, Aberdeen AB25 2ZD, UK; 2 Biomathematics & Statistics Scotland, Rowett Institute, University of Aberdeen, Foresterhill Health Campus, Aberdeen AB25 2ZD, UK; 3 Department of Infectious Disease Epidemiology, London School of Hygiene & Tropical Medicine, Keppel Street, London WC1E 7HT, UK; 4 Division of Microbiology, National Institute of Health Sciences, 3-25-26 Tonomachi, Kawasaki-ku, Kawasaki-shi 210-9501, Kanagawa, Japan; 5 Scottish Organic Producers Association (SOPA), Perth PH2 8BX, UK

**Keywords:** *Fusarium* mycotoxins, trichothecenes, masked mycotoxins, organic, conventional, oats

## Abstract

Small grain cereals are frequently infected with mycotoxigenic *Fusarium* fungi. Oats have a particularly high risk of contamination with type A trichothecene mycotoxins; their glucoside conjugates have also been reported. Agronomy practices, cereal variety and weather conditions have been suggested to play a role in *Fusarium* infection in oats. The current study investigates concentrations of free and conjugated *Fusarium* mycotoxins in organic and conventional oats grown in Scotland. In 2019, 33 milling oat samples (12 organic, 21 conventional) were collected from farmers across Scotland, together with sample questionnaires. Samples were analysed for 12 mycotoxins (type A trichothecenes T-2-toxin, HT-2-toxin, diacetoxyscirpenol; type B trichothecenes deoxynivalenol, nivalenol; zearalenone and their respective glucosides) using LC-MS/MS. The prevalence of type A trichothecenes T-2/HT-2 was very high (100% of conventional oats, 83% of organic oats), whereas type B trichothecenes were less prevalent, and zearalenone was rarely found. T-2-glucoside and deoxynivalenol-glucoside were the most prevalent conjugated mycotoxins (36 and 33%), and co-occurrence between type A and B trichothecenes were frequently observed (66% of samples). Organic oats were contaminated at significantly lower average concentrations than conventional oats, whereas the effect of weather parameters were not statistically significant. Our results clearly indicate that free and conjugated T-2- and HT-2-toxins pose a major risk to Scottish oat production and that organic production and crop rotation offer potential mitigation strategies.

## 1. Introduction

Fungal infection is a major problem in global cereal production and results in subsequent contamination of grains with a wide range of mycotoxins. In temperate regions, *Fusarium* is the predominant mycotoxigenic genus found to infect small grain cereals in the field pre-harvest [1,2]. Prominent strains within the genus *Fusarium* include *F. graminearum*, *F. culmorum*, *F. langsethiae* and *F. poae*, all of which have been shown to produce a range of mycotoxins including trichothecenes (type A and B) and zearalenone in small grain cereals [3,4,5]. Type A trichothecenes include potent immunotoxins and intestinal toxins T-2-toxin (T-2), HT-2-toxin (HT-2) and diacetoxyscirpenol (DAS), while type B trichothecenes include deoxynivalenol (DON) and nivalenol (NIV) [6]. Both trichothecenes and zearalenone (ZEN) have been reported in small grain cereals, including wheat [7,8,9,10,11], barley [12,13,14,15] and oats [12,16,17,18,19] grown in temperate regions of Europe and North America. Based on their varying toxicity, a range of regulatory limits are set in Europe to minimise human exposure and manage potential risks to consumers (Table 1).

Recent UK surveys have identified T-2/HT-2 to occur commonly in food oats, although exceedances of European Commission (EC) indicative levels are rare [22,23]. In addition to the free fungal mycotoxins, plant-derived modified mycotoxins such as sugar-conjugated forms DON-glucoside and ZEN-glucoside have also been reported in wheat [12,24,25,26] at proportions of 4–69% of the free parent mycotoxins. Conjugated glucoside forms of T-2 and HT-2 have also been identified [27], but less information is available on their natural occurrence in cereal grains. These conjugated mycotoxins are released by the activity of the intestinal microbiota in vitro [27,28,29,30,31,32,33] and have been found to contribute to human exposure to free mycotoxins in vivo [34]. Hence the presence of free and modified mycotoxins in cereals warrants further investigation. 

Previous studies have identified a range of agronomy practices that might decrease the risk of fungal infection and mycotoxin contamination in cereals. These include spring rather than winter sowing, varietal selection (for wheat) and cereal rotation [35,36,37,38]. Furthermore, some studies suggest that organic production systems may lower mycotoxin contamination in some cereals [39]. Hence, the current paper presents a detailed profiling of free and sugar-conjugated *Fusarium* mycotoxins in oat samples grown in conventional or organic systems in Scotland. 

## 2. Results

### 2.1. Prevalence of Free and Modified Mycotoxins in Organic and Conventional Oats 

**Figure 1:**
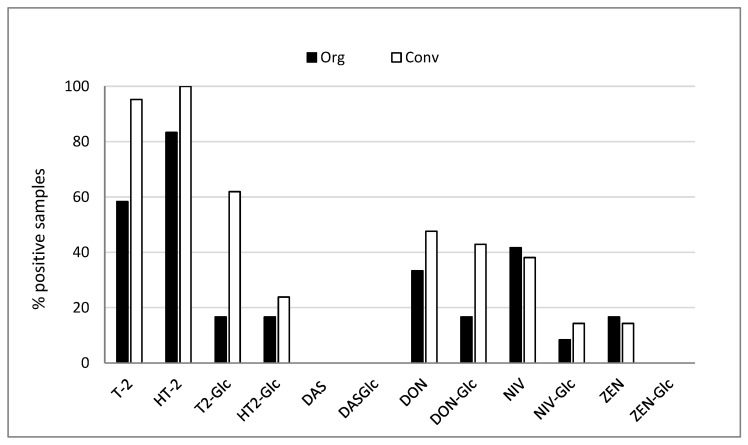
Prevalence of mycotoxins in organic (n = 12) and conventional (n = 21) oat samples. Data are presented as percentage of samples contaminated >LOQ for each mycotoxin. LOQ = limit of quantification.

Type A trichothecenes T-2 and HT-2 were highly prevalent in Scottish oat samples, with higher prevalence observed in conventional oats (95.2 and 100%, respectively), compared to organic oats (58.3 and 83.3%, respectively. In contrast, DAS and DAS-Glc were not detected (Figure 1). 

Overall, type B trichothecenes DON and NIV were less prevalent in oats than type A trichothecenes. The difference between organic and conventional oats was less pronounced (DON 33.3 and 47.6; NIV 41.7 and 38.1%). T-2-Glc and DON-Glc were the most prevalent modified mycotoxins, especially in conventional oats (61.9 and 42.8%, respectively). ZEN was not frequently detected, and no ZEN-Glc was found in any sample.

### 2.2. Concentrations of Free and Modified Mycotoxins in Organic and Conventional Oats

**Figure 2:**
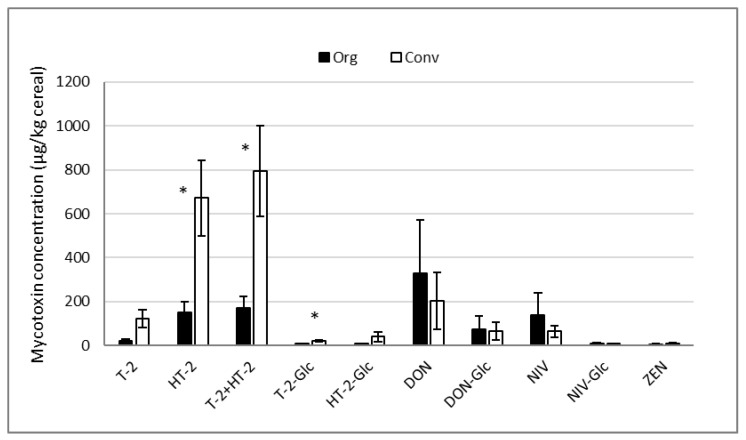
Average concentrations of mycotoxins in organic (n = 12) and conventional (n = 21) oat samples. Data are presented as mean concentration ± SEM, and data points < LOQ were replaced by values of ½ of LOQ [40]. * Indicates significant (*p* < 0.05) difference between organic and conventional oats.

HT-2 in conventional oats was the highest mycotoxin concentration found in any sample group in this study (average 670.8 µg/kg, Figure 2). In addition, T-2 and HT-2 toxins were frequently found in the same sample resulting in 19% of conventional oat samples exceeding the EC indicative level of 1000 µg/kg for T-2 + HT-2. However, concentrations of T-2 and HT-2 in organic oat samples were significantly lower (21.1 and 148.9 µg/kg mean concentration, *p* = 0.0023 and *p* = 0.0043, respectively), with no organic oats exceeding the EC indicative level.

DON concentrations were not significantly different (*p* = 0.9828) between organic and conventional oats (mean 327.3 and 204.0 µg/kg, respectively), with 1/12 organic and 1/21 conventional samples exceeding the EC maximum permitted level for DON (1750 µg/kg). T-2-Glc was the most frequently detected modified mycotoxin in conventional oats (61.9% prevalence, Figure 1) at ratios ranging from 6–154% of T-2 (Table 2). Ratios of HT-2-Glc ranged from 34–174% of HT-2, whereas DON-Glc was found at lower ratios (18–130% of DON).

### 2.3. Co-Occurrence of Free Mycotoxins in Organic and Conventional Oats

Oat samples were frequently contaminated with numerous mycotoxins in different combinations. Co-occurrence is defined here as the presence of type A trichothecenes (T-2/HT-2), type B trichothecenes (DON or NIV) and ZEN. Modified mycotoxins are not included in these figures as they represent plant metabolites of the parent mycotoxins produced by fungi. One conventional oat sample (4.8%) was co-contaminated with all four mycotoxins, while two or more mycotoxins co-occurred in 50.0% organic oats and 61.9% conventional oat samples (Table 3). None of the samples in this survey were free of all mycotoxins tested (i.e., all mycotoxins < LOQ). 

**Figure 3:**
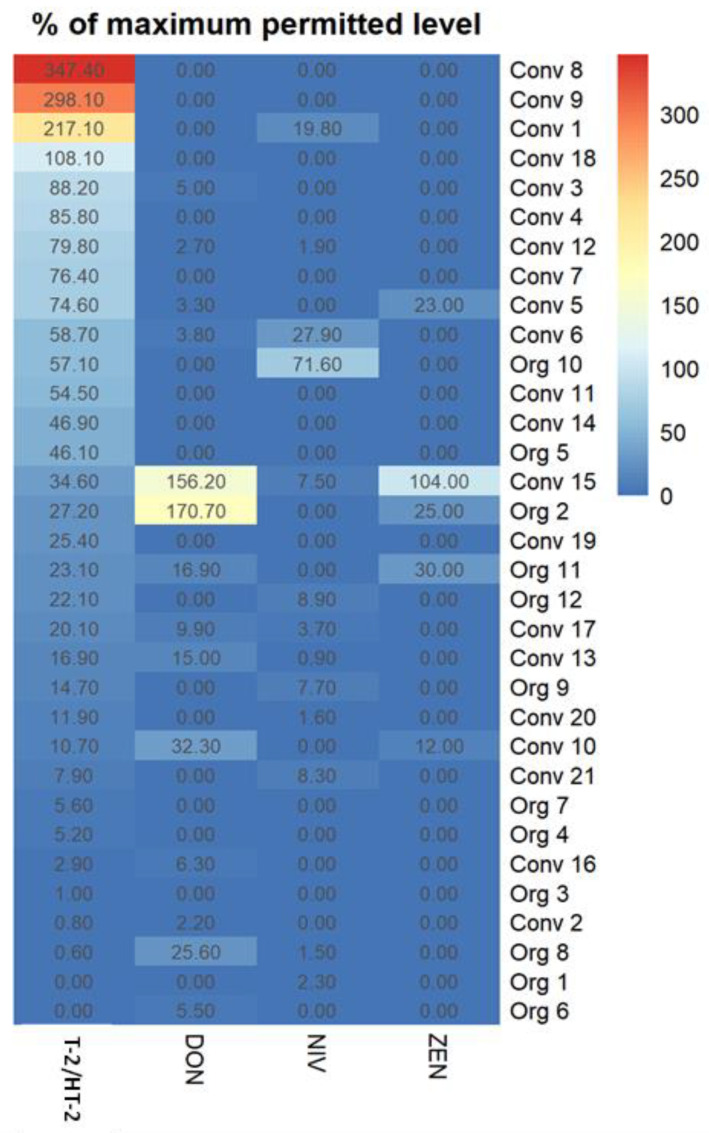
Heatmap depicting co-occurrence of multiple free mycotoxins in individual oat samples. Mycotoxin levels are expressed as % of the EC indicative level for T-2/HT-2, % of the EC maximum permitted level for DON (used for DON and NIV), and % of the EC maximum permitted level for ZEN in unprocessed oats (Table 1). The heatmap was generated in “R” [version 4.2.1 (2022-06-23)], Org = organic oat sample, conv = conventional oat sample.

The highest concentrations of T-2/HT-2 (>100% of EC indicative levels) were found in four conventional oats, but these samples were not heavily co-contaminated with type B trichothecenes or ZEN (Figure 3). Conversely, samples with the highest levels of DON were also co-contaminated with ZEN. 

### 2.4. Effect of Other Agronomy Factors on Mycotoxin Concentrations in Oats

**Figure 4:**
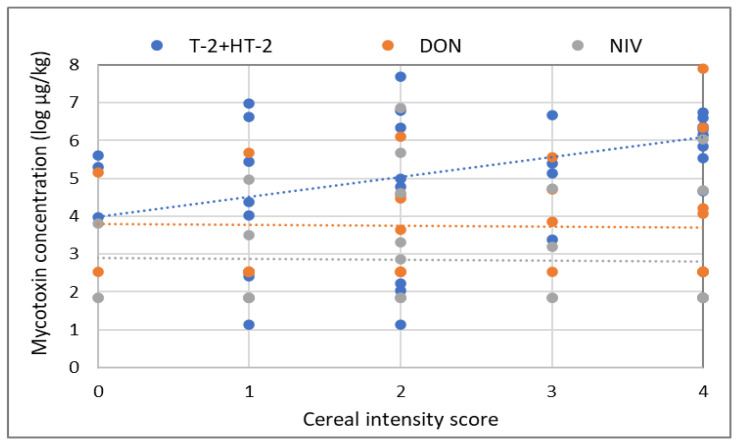
Relationship between cereal intensity score and mycotoxin concentration across all samples in this survey. The data are log-transformed, and the lines show the fitted model for each mycotoxin. Trendlines for relationships are added for T-2 + HT-2 (blue), DON (orange) and NIV (grey).

In addition to organic versus conventional oat production, the effect of cereal rotation intensity and weather conditions, such as average monthly rainfall and average monthly temperature one month and two months prior to harvest date, were investigated for their potential impact on mycotoxin concentrations. Cropping history was examined, and a cereal intensity score was calculated [40] as the number of years (over the previous 4 years) during which the previous crop was a small grain cereal (wheat, oats or barley). A significant positive relationship (*p* = 0.0426) was found between the cereal intensity score and the levels of T-2/HT-2 across all oat samples in this survey (Figure 4). No other significant relationships were found between mycotoxin levels and other factors in this dataset.

## 3. Discussion

Type A trichothecene mycotoxins T-2/HT-2 are well-recognised as major contaminants in oat production [17,39,40,41]. Our study confirms that these mycotoxins occur at the highest prevalence and concentration in this Scottish sample set. Additionally, our survey demonstrates the high prevalence of the modified mycotoxins T-2-Glc and HT-2-Glc in oat samples, further increasing the overall contamination levels. In a longitudinal survey of mycotoxins in UK cereal production [42], authors report a prevalence of T-2/HT-2 of 86–100% (29 food oat samples each year) with mean concentrations of 313–458 µg/kg sample. The prevalence of contamination is comparable to our study (100% prevalence in conventional oats), but mean concentrations are higher in our Scottish survey (793 µg/kg). There are currently no maximum regulatory levels set for T-2/HT-2 in oats, but indicative levels can be used to benchmark contamination levels. In our survey, 19% of conventional oats and no organic oats exceeded the EC indicative level for T-2/HT-2, resulting in overall 12% exceedances across all 33 oat samples, which are comparable to other studies reporting 1–30% exceedances in conventional oats in the UK [40] and 7.4% exceedances in organic and conventional oats in Ireland [43].

The prevalence and mean concentration of T-2-Glc reported in the AHDB survey [42] (59–79%, 37.1–67.4 µg/kg) are also similar to our results (61.9%, 21.9 µg/kg), but we also detected HT-2-Glc in 24% of samples (mean 39.2 µg/kg) which were not assessed in previous studies. Furthermore, despite the low mean concentration across the samples, we observed that two conventional oat samples contained high levels of HT-2-Glc (462 and 155 µg/kg), which significantly contributes to the overall mycotoxin contamination of these samples. Previous in vitro studies have clearly shown that T-2-Glc and HT-2-Glc are rapidly hydrolysed to free T-2 and HT-2 by the microbial activity of the human gut microbiota [27,31] and can therefore contribute to overall exposure to these potent mycotoxins in humans. Hence further investigations into the levels of modified forms of T-2/HT-2 in unprocessed cereals and their carry-over into food products are needed.

Organic oats have previously been found to be contaminated with lower levels of T-2/HT-2 compared to conventional oats in studies conducted in the UK [17,40], Ireland [43], Norway, Poland and Germany [39], while no such consistent differences were found in other cereals [39]. Similarly, we found the T-2/HT-2 levels to be significantly lower in organic oat samples compared to conventional oats. Furthermore, we also found T-2-Glc + HT-2-Glc to be significantly lower in organic oats, further supporting the notion that organic production can decrease the risk of mycotoxin contamination in oats.

Other agronomic factors have also been identified to impact the risk of fungal infection and mycotoxin contamination in cereals. Among them, cereal rotations, ploughing and sowing dates (winter versus spring sowing) have been found to be important factors affecting oat mycotoxin concentrations [40,43,44]. In the present study, we could also confirm that cereal intensity increased the risk of T-2/HT-2 contamination but not other mycotoxins. Similarly, Kolawole et al. (2021) report that previous crops have a stronger impact on T-2/HT-2 than on DON and ZEN contamination in oats [43], and Edwards (2017) reports that cereal intensity was significantly related to T-2/HT-2 levels. Still, DON and ZEN were not investigated as they were detected less frequently in UK oats [40]. These published studies also demonstrate that crop growth season is an important factor, with spring-sown oats containing significantly lower concentrations of mycotoxins than winter-sown [40,43]. However, we were unable to assess the effect of crop growth season as only one sample in the current survey was winter-sown. 

In summary, this study clearly demonstrates the high prevalence of type A trichothecenes in Scottish oat samples and the frequent co-contamination with type B trichothecenes and zearalenone. In addition, the study indicates the protective effect of organic agronomy against high mycotoxin contamination and points towards the potential benefits of low-intensity cereal rotations.

## 4. Materials and Methods

### 4.1. Study Design

This study was carried out in collaboration with SOPA, Farmton Farm, WN Lindsay and Hamlyns of Scotland. The collaborators designed a detailed sample questionnaire (Supplementary Materials) approved by the Rowett Institute Human Studies Ethics Committee (16 July 2019). Farmers were approached through links with the project collaborators and were asked to provide a 1 kg aggregate sample of unprocessed, dried (<14% moisture content) milling oats. Farmers were asked to complete the sample questionnaire as paper copies or using the online tool https://tinyurl.com. (accessed on 20 August 2019) In total, 33 oat samples and corresponding questionnaires were obtained, including 12 samples from organic farms and 21 from conventional farms and were stored at room temperature at the Rowett Institute. Fewer organic than conventional samples reflects the balance between organic and conventional cropping in Scotland. Information on organic status, fungicide use, crop rotation practices, oat variety and harvest date were obtained from sample questionnaires. Total monthly rainfall (mm) and average monthly temperature (degrees centigrade) for the (1 month prior = pre-harvest period, 2 months prior = flowering) period prior to harvest were obtained from the Met Office weather survey for the area of each farm (https://www.metoffice.gov.uk/research/climate/maps-and-data/uk-actual-and-anomaly-maps, accessed on 29 October 2020). 

### 4.2. Mycotoxin Determination in Oat Samples

#### 4.2.1. Mycotoxin Standards

T-2-toxin (T-2), HT-2-toxin (HT-2), diacetoxyscirpenol (DAS), [^13^C_22_] HT-2, deoxynivalenol (DON), [^13^C_15_] DON, DON-3-β,D-glucoside (DON-Glc), nivalenol (NIV), zearalenone (ZEN) and [^13^C_18_] ZEN, were purchased from Romer Labs Ltd., Tulln, Austria. DAS-3-α, D-glucoside (DAS-Glc), T-2-3-α,D-glucoside (T-2-Glc) [45] and HT-2-3-β,D-glucoside (HT-2-Glc) [46] were obtained from Dr. Mark Busman and Dr Susan McCormick, Mycotoxin Prevention and Applied Microbiology Unit, USDA-ARS-NCAUR in the USA. NIV-3-β,D-glucoside (NIV-Glc) was obtained from Dr. Tomoya Yoshinari, National Institute of Health Sciences, Japan [47]. ZEN-14-β,D-glucoside (ZEN-Glc) standard used in this study was previously synthesised as part of FSA-funded project FS102101. Working solutions for all mycotoxins were prepared in acetonitrile (ACN) and stored at 4 °C (Table 4). 

Eight-point calibration curves (DON 0.625–500 ng/mL; HT-2 0.3125–250 ng/mL DON-Glc, NIV, NIV-Glc, T-2, T-2-Glc, HT-2-Glc, ZEN, ZEN-Glc 0.1563–125 ng/mL) were used to quantify all analytes. Stable-isotope labelled internal standards were used as follows: DON ^13^C_15_ (50 ng/mL) was used to quantify DON, HT-2 ^13^C_22_ (50 ng/mL) was used to quantify HT-2 and T-2, and ZEN ^13^C_18_ (25 ng/mL) was used to quantify ZEN. For other mycotoxins and modified mycotoxins (DON-Glc, NIV, NIV-Glc, DAS, DAS-Glc, T-2-Glc, HT-2-Glc, ZEN-Glc), external calibration curves were used in quantification.

#### 4.2.2. Extraction of Oat Samples

Oat samples were freeze-milled by using a 6870 large freezer/Mill (SPEX SamplePrep, Metuchen, NJ, USA) into fine powder. Next, 0.5 g milled and homogenised oat samples were extracted with 2 mL of extraction solvent (79% ACN, 20% H_2_O, 1% acetic acid; HAc) [48] for 90 min at 1200 rpm on an orbital shaker (IKA^®^ VXR basic, Thomson Scientific, Aberdeen, UK). Samples were centrifuged at room temperature (2000× *g* for 5 min), and supernatants were dried under nitrogen stream and reconstituted to achieve 10% of ACN in sample extracts. Prior to LC-MS/MS analysis, sample extracts were combined with ^13^C_22_-HT-2, ^13^C_15_-DON and ^13^C_18_-ZEN to facilitate the quantification of parent mycotoxins using a stable isotope dilution approach (SIDA) [49].

#### 4.2.3. LC-MS/MS Analysis of Mycotoxins

The detection and the quantification of all mycotoxins and the [^13^C]-labelled standards were performed on a Shimadzu Nexera X2 LC Quaternary pump coupled to a Shimadzu 8060 mass spectrometer fitted with an electrospray ionisation (ESI) source (Shimadzu, Kyoto, Japan). The liquid chromatography separation was performed on a Phenomenex Gemini C18 column, 150 mm × 3 mm, particle size 3 µm. Mobile phase solvents were (A) 0.1% HAc and (B) methanol; after 2 min at 100% A, the proportion of B was increased linearly to 100% within 12 min, followed by a hold time of 3 min at 100% B and 4 min column re-equilibration at 100% A. The flow rate was 800 µL/min, and the injection volume was 15 µL. The LC eluent was directed into the ESI source without splitting. The mass spectrometer was run in positive and negative ion mode with the following settings: interface temperature 300 °C, desolvation temperature 250 °C, heating block temperature 300 °C, and gases 1 and 2 set at 15 and 5 L/min, respectively. Argon gas was used as the collision gas in the collision cell for the fragmentation of the mycotoxin metabolites. Ion transition parameters and precursors used for each mycotoxin are summarised in Table 4. Mycotoxins were quantified using the multiple reaction monitoring (MRM) technique. Standard solutions of approximately 1 ng/µL concentration were prepared and put into the LC auto sampler, where the mass spectrometer sampled from them automatically to optimise the MRM conditions of the individual mycotoxin metabolites.

### 4.3. Method Performance Validation

Performance characterisation included absolute recovery (RA), signal suppression/enhancement (SSE) and limit of quantification (LOQ). Recovery was assessed in triplicate by spiking a blank oats sample (0.5 g) with a mycotoxin mix (15 µL in acetonitrile) containing 300 µg/kg DON, 150 µg/kg HT-2 and 75 µg/kg DON-Glc, NIV, NIV-Glc, T-2, T-2-Glc, HT-2-Glc, DAS, DAS-Glc, ZEN, ZEN-Glc. Following evaporation (37 °C, 30 min), samples were extracted as described above (section: extraction of oat samples). Absolute recovery (RA) [50] was calculated as

RA (%) = Observed concentration in spike sample/Spike concentration × 100

The matrix-matched calibration curves (8 levels, in triplicate) were prepared in blank oat extracts and compared to solvent calibration curves to calculate signal suppression/enhancement (SSE%) as

SSE (%) = matrix-matched calibration curves slope/solvent calibration curves slope × 100.

LOQ was determined in oat matrix by a signal-to-noise ratio of 10/1.

### 4.4. Data calculations and Statistical Analysis

All results were corrected for recovery. For prevalence (% of positive samples), only values > LOQ for each mycotoxin were included. For calculation of mean concentration of mycotoxins and statistical analysis, all values < LOQ for each mycotoxin were replaced by ½ LOQ [40]

Mycotoxin concentrations were log-transformed, and for each mycotoxin (single toxins T-2, HT-2, T-2-Glc, HT-2-Glc, DON, DON-Glc, NIV, NIV-Glc as well as T-2 + HT-2 and T-2 + HT-2 + T-2-Glc + HT-2Glc) were fitted to covariates (organic production, cereal intensity score, cereal variety, temperature, rainfall and harvest date) using linear models. Due to sample size, models with one or two covariates were fitted at a time. Analysis of variance was used to test the statistical significance of the covariates in each model, with *p* < 0.05 considered significant. Diagnostics were carried out to assess the assumptions of the tests. All analyses were carried out using the statistical software “R”, version 4.2.2 (R Core Team, 2021. R: A language and environment for statistical computing. R Foundation for Statistical Computing, Vienna, Austria).

## Figures and Tables

**Table 1:** Overview of EC maximum levels of selected mycotoxins in oat products.

Mycotoxin	Oat Product	Maximum Level (µg/kg)
T-2 + HT-2 ^1^	Unprocessed oats	1000
	Oat grains for direct human consumption	200
	Oat bran and flakes	200
DON ^2^	Unprocessed oats	1750
	Oats intended for direct human consumption, oat flour, oat meal, oat bran, or germ	750
	Bread, pastries, biscuits, cereal snacks and breakfast cereals	500
ZEN ^2^	Unprocessed oats	100
	Oats intended for direct human consumption, oat flour, oat meal, oat bran or germ	75
	Bread, pastries, biscuits, cereal snacks and breakfast cereals	50

^1^ for T-2 + HT-2 indicative levels are set by EC recommendation 2013/165/EU [20]. ^2^ for DON and ZEN maximum permitted levels are set by EC regulation 1881/2006 [21].

**Table 2:** Free and modified mycotoxins in organic and conventional oat samples.

Oat ID	T-2 µg/kg	HT-2 µg/kg	T-2-Glc µg/kg	HT-2-Glc µg/kg	T-2-Glc %	HT-2-Glc %	DON µg/kg	NIV µg/kg	DON-Glc µg/kg	NIV-Glc µg/kg	DON-Glc %	NIV-Glc %
Org1	ND	ND	ND	19	---	---	ND	41	ND	ND	---	---
Org2	42	230	ND	ND	---	---	2988	ND	746	ND	25	---
Org4	ND	52	ND	ND	---	---	ND	ND	ND	ND	---	---
Org5	71	390	11	ND	16	---	ND	ND	ND	ND	---	---
Org6	ND	ND	ND	ND	---	---	97	ND	ND	ND	---	---
Org7	3	53	ND	ND	---	---	ND	ND	ND	ND	---	---
Org8	ND	6	ND	ND	---	---	448	27	ND	ND	---	---
Org9	9	138	ND	ND	---	---	ND	134	ND	ND	---	---
Org10	33	538	18	ND	55	---	ND	1253	ND	44	---	4
Org11	58	173	ND	ND	---	---	295	ND	84	ND	28	---
Org12	28	193	ND	ND	---	---	ND	156	ND	ND	---	---
Conv1	266	1905	ND	ND	---		ND	346	ND	20	---	6
Conv2	ND	8	ND	13	---	174	38	ND	50	ND	130	---
Conv3	33	849	51	ND	154	---	88	ND	57	ND	65	---
Conv4	88	770	27	ND	31	---	ND	ND	ND	ND	---	---
Conv5	97	649	23	ND	24	---	58	ND	ND	ND	---	---
Conv6	67	520	ND	ND	---	---	67	488	15	24	23	5
Conv7	155	609	28	ND	18	---	ND	ND	ND	ND	---	---
Conv8	390	3084	31	ND	8	---	ND	ND	28	ND	---	---
Conv9	836	2145	53	ND	6	---	ND	ND	ND	ND	---	---
Conv10	41	66	ND	ND	---	---	565	ND	101	ND	18	---
Conv11	36	509	47	ND	130	---	ND	ND	ND	ND	---	---
Conv12	93	705	73	ND	78	---	48	33	ND	ND	---	---
Conv13	32	137	44	ND	137	---	263	15	47	ND	18	---
Conv14	126	343	14	ND	11	---	ND	ND	ND	ND	---	---
Conv15	48	298	ND	ND	---	---	2734	132	881	21	32	16
Conv16	3	26	ND	ND	---	---	111	ND	ND	ND	---	---
Conv17	44	157	ND	ND	---	---	173	64	89	ND	51	---
Conv18	167	914	ND	462	---	51	ND	ND	ND	ND	---	---
Conv19	34	220	ND	155	---	70	ND	ND	ND	ND	---	---
Conv20	22	97	ND	67	---	70	ND	28	ND	ND	---	---
Conv21	4	75	ND	26	---	34	ND	145	29	ND	---	---

Org = organic oat sample, Conv = conventional oat sample, ND = not detected (<LOQ).

**Table 3:** Co-occurrence of free mycotoxins in organic and conventional oats.

Number of Co-Occurring Mycotoxins	Number of Combinations Found	Types of Combinations	Number (%) of Samples Organic	Number (%) of Samples Conventional
4	1	T-2/HT-2 + DON + NIV + ZEN	0 (0)	1 (4.8)
3	2	T-2/HT-2 + DON + NIV T-2/HT-2 + DON + ZEN	1 (8.3) 2 (16.7)	4 (19.0) 2 (9.5)
2	2	T-2/HT-2 + DON T-2/HT-2 + NIV	0 (0) 3 (25.0)	3 (14.3) 3 (14.3)
(1)	3	T-2/HT-2 DON NIV	4 (33.3) 1 (8.3) 1 (8.3)	8 (38.1) 0 (0) 0 (0)

**Table 4:** Summary of LC-MS/MS parameters and method performance parameters for all mycotoxins used.

Compound	RT (min)	Precursor Ion (m/z)	Product Ion (m/z)	Collision Energy	Polarity	% RA (RSD)	%SSE (RSD)	LOQ Oat
T-2	10.3	489.1	327.2	−26.0	+ve	109.4 (2.2)	103.3 (8.4)	3.1
HT-2	9.8	447.3	345.2	−20.0	+ve	92.3 (6.8)	91.4 (9.1)	6.3
T-2-Glc	9.9	651.3	489.2	−34.0	+ve	118.7 (3.6)	96.5 (3.3)	12.5
HT-2-Glc	9.4	609.2	447.1	−34.0	+ve	114.1 (5.7)	77.4 (8.8)	12.5
DAS	8.9	384.2	307.5	−12.0	+ve	95.3 (16.3)	116.5 (5.0)	25
DAS-Glc	8.5	551.2	389.1	−33.0	+ve	112.0 (7.1)	111.7 (1.8)	25
DON	6.1	355.3	295.2	12.0	−ve	87.9 (7.6)	107.3 (4.9)	25
DON-Glc	5.9	517.2	427.2	23.0	−ve	95.1 (10.6)	93.2 (6.4)	12.5
NIV	5.3	371.2	281.2	20.0	−ve	101.1 (7.3)	93.3 (7.4)	12.5
NIV-Glc	5.1	533.3	473.2	14.0	−ve	94.7 (14.5)	91.9 (2.8)	12.5
ZEN	10.9	317.2	175.3	24.0	−ve	86.4 (6.4)	104.4 (7.9)	6.3
ZEN-Glc	9.4	479.4	317.2	21.0	−ve	70.4 (8.1)	59.6 (5.0)	6.3
^13^C_22_-HT-2	9.8	464.3	278.2	−20.0	+ve			
^13^C_15_-DON	6.1	370.2	310.3	11.0	−ve			
^13^C_18_-ZEN	10.9	335.2	185.2	26.0	−ve			

## Data Availability

The data presented in this study are available on request from the corresponding author. The data are not publicly available due to industry collaboration.

## Supplementary Materials

The sample questionnaire can be downloaded at: https://www.mdpi.com/article/10.3390/toxins15040247/s1, Farm Agronomy Questionnaire.

## References

[1] Tran M.T., Ameye M., Phan L.T., Devlieghere F., De Saeger S., Eeckhout M., Audenaert K. (2021). Impact of Ethnic Pre-Harvest Practices on the Occurrence of *Fusarium Verticillioides* and Fumonisin B1 in Maize Fields from Vietnam. Food Control.

[2] Degraeve S., Madege R., Audenaert K., Kamala A., Ortiz J., Kimanya M., Tiisekwa B., De Meulenaer B., Haesaert G. (2016). Impact of Local Pre-Harvest Management Practices in Maize on the Occurrence of *Fusarium* Species and Associated Mycotoxins in Two Agro-Ecosystems in Tanzania. Food Control.

[3] Pasquali M., Beyer M., Logrieco A., Audenaert K., Balmas V., Basler R., Boutigny A., Chrpova J., Czembor E., Gagkaeva T. (2016). A European Database of Fusarium Graminearum and *F. Culmorum* Trichothecene Genotypes. Front. Microbiol..

[4] Hofgaard I.S., Aamot H.U., Seehusen T., Riley H., Dill-Macky R., Holen B., Brodal G. (2020). Fusarium and Mycotoxin Content of Harvested Grain was Not Related to Tillage Intensity in Norwegian Spring Wheat Fields. World Mycotoxin J..

[5] Fredlund E., Gidlund A., Sulyok M., Börjesson T., Krska R., Olsen M., Lindblad M. (2013). Deoxynivalenol and Other Selected Fusarium Toxins in Swedish oats—Occurrence and Correlation to Specific *Fusarium* Species. Int. J. Food Microbiol..

[6] Polak-śliwińska M., Paszczyk B. (2021). Trichothecenes in Food and Feed, Relevance to Human and Animal Health and Methods of Detection: A Systematic Review. Molecules.

[7] Alkadri D., Rubert J., Prodi A., Pisi A., Manes J., Soler C. (2014). Natural Co-Occurrence of Mycotoxins in Wheat Grains from Italy and Syria. Food Chem..

[8] Schollenberger M., Jara H.T., Suchy S., Drochner W., Müller H.-M. (2002). *Fusarium* Toxins in Wheat Flour Collected in an Area in Southwest Germany. Int. J. Food Microbiol..

[9] Rasmussen P.H., Ghorbani F., Berg T. (2003). Deoxynivalenol and Other *Fusarium* Toxins in Wheat and Rye Flours on the Danish Market. Food Addit. Contam..

[10] Hajšlová J., Lancová K., Sehnalová M., Krplová A., Zachariášová M., Moravcová H., Nedělník J., Marková J., Ehrenbergerová J. (2007). Occurrence of Trichothecene Mycotoxins in Cereals Harvested in the Czech Republic. Czech J. Food Sci..

[11] Edwards S.G. (2009). *Fusarium* Mycotoxin Content of UK Organic and Conventional Wheat. Food Addit. Contam. Part A Chem. Anal. Control Expo. Risk Assess..

[12] Nathanail A.V., Syvahuoko J., Malachova A., Jestoi M., Varga E., Michlmayr H., Adam G., Sievilainen E., Berthiller F., Peltonen K. (2015). Simultaneous Determination of Major Type A and B Trichothecenes, Zearalenone and Certain Modified Metabolites in Finnish Cereal Grains with a Novel Liquid Chromatography-Tandem Mass Spectrometric Method. Anal. Bioanal. Chem..

[13] Edwards S.G. (2009). *Fusarium* Mycotoxin Content of UK Organic and Conventional Barley. Food Addit. Contam. Part A Chem. Anal. Control Expo. Risk Assess..

[14] Barthel J., Gottschalk C., Rapp M., Berger M., Bauer J., Meyer K. (2012). Occurrence of Type A, B and D Trichothecenes in Barley and Barley Products from the Bavarian Market. Mycotoxin Res..

[15] Drakopoulos D., Sulyok M., Krska R., Logrieco A.F., Vogelgsang S. (2021). Raised Concerns about the Safety of Barley Grains and Straw: A Swiss Survey Reveals a High Diversity of Mycotoxins and Other Fungal Metabolites. Food Control.

[16] Ivanova L., Sahlstrom S., Rud I., Uhlig S., Faeste C.K., Eriksen G.S., Divon H.H. (2017). Effect of Primary Processing on the Distribution of Free and Modified *Fusarium* Mycotoxins in Naturally Contaminated Oats. World Mycotoxin J..

[17] Edwards S.G. (2009). *Fusarium* Mycotoxin Content of UK Organic and Conventional Oats. Food Addit. Contam. Part A Chem. Anal. Control Expo. Risk Assess..

[18] Meyer J.C., Hennies I., Wessels D., Schwarz K. (2021). Survey of Mycotoxins in Milling Oats Dedicated for Food Purposes between 2013 and 2019 by LC–MS/MS. Food Addit. Contam. Part A Chem. Anal. Control Expo. Risk Assess..

[19] Tarazona A., Gómez J.V., Mateo F., Jiménez M., Mateo E.M. (2021). Potential Health Risk Associated with Mycotoxins in Oat Grains Consumed in Spain. Toxins.

[20] EC—European Commission (2013). Commission Recommendation of 27 March 2013 on the Presence of T-2 and HT-2 Toxin in Cereals and Cereal Products. Off. J. Eur. Comm. L.

[21] European Commission (2006). Commission Regulation (EC) no 1881/2006 of 19 December 2006 Setting Maximum Levels for Certain Contaminants in Foodstuffs. Off. J. Eur. Union..

[22] Byrd N., Slaiding I.R. (2016). Monitoring of Mycotoxins and Other Contaminants in UK Cereals used in Malting, Milling & Animal Feed. FERA.

[23] (2022). Monitoring of Contaminants in UK Cereals used for Processing Food and Animal Feed (2016–22).

[24] De Boevre M., Di Mavungu J.D., Maene P., Audenaert K., Deforce D., Haesaert G., Eeckhout M., Callebaut A., Berthiller F., Van Peteghem C. (2012). Development and Validation of an LC-MS/MS Method for the Simultaneous Determination of Deoxynivalenol, Zearalenone, T-2-Toxin and some Masked Metabolites in Different Cereals and Cereal-Derived Food. Food Addit. Contam. Part A Chem. Anal. Control Expo. Risk Assess..

[25] Rasmussen P.H., Nielsen K.F., Ghorbani F., Spliid N.H., Nielsen G.C., Jørgensen L.N. (2012). Occurrence of Different Trichothecenes and Deoxynivalenol-3-Β-D-Glucoside in Naturally and Artificially Contaminated Danish Cereal Grains and Whole Maize Plants. Mycotoxin Res..

[26] Bryla M., Ksieniewicz-Wozniak E., Waskiewicz A., Szymczyk K., Jedrzejczak R. (2018). Natural Occurrence of Nivalenol, Deoxynivalenol, and Deoxynivalenol-3-Glucoside in Polish Winter Wheat. Toxins.

[27] McCormick S.P., Kato T., Maragos C.M., Busman M., Lattanzio V.M.T., Galaverna G., Dall-Asta C., Crich D., Price N.P.J., Kurtzman C.P. (2015). Anomericity of T-2 Toxin-Glucoside: Masked Mycotoxin in Cereal Crops. J. Agric. Food Chem..

[28] Gratz S.W., Duncan G., Richardson A.J. (2013). The Human Fecal Microbiota Metabolizes Deoxynivalenol and Deoxynivalenol-3-Glucoside and may be Responsible for Urinary Deepoxy-Deoxynivalenol. Appl. Environ. Microbiol..

[29] Dall’Erta A., Cirlini M., Dall’Asta M., Del Rio D., Galaverna G., Dall’Asta C. (2013). Masked Mycotoxins are Efficiently Hydrolyzed by Human Colonic Microbiota Releasing their Aglycones. Chem. Res. Toxicol..

[30] Gratz S.W., Dinesh R., Yoshinari T., Holtrop G., Richardson A.J., Duncan G., Macdonald S., Lloyd A., Tarbin J. (2017). Masked Trichothecene and Zearalenone Mycotoxins Withstand Digestion and Absorption in the Upper GI Tract but are Efficiently Hydrolyzed by Human Gut Microbiota in Vitro. Mol. Nutr. Food Res..

[31] Daud N., Currie V., Duncan G., Busman M., Gratz S.W. (2020). Intestinal Hydrolysis and Microbial Biotransformation of Diacetoxyscirpenol-Alpha-Glucoside, HT-2-Beta-Glucoside and N-(1-Deoxy-D-Fructos-1-Yl) Fumonisin B-1 by Human Gut Microbiota in Vitro. Int. J. Food Sci. Nutr..

[32] Daud N., Currie V., Duncan G., Farquharson F., Yoshinari T., Louis P., Gratz S.W. (2020). Prevalent Human Gut Bacteria Hydrolyse and Metabolise Important Food-Derived Mycotoxins and Masked Mycotoxins. Toxins.

[33] Gratz S.W., Currie V., Richardson A.J., Duncan G., Holtrop G., Farquharson F., Louis P., Pinton P., Oswald I.P. (2018). Porcine Small and Large Intestinal Microbiota Rapidly Hydrolyze the Masked Mycotoxin Deoxynivalenol-3-Glucoside and Release Deoxynivalenol in Spiked Batch Cultures in Vitro. Appl. Environ. Microbiol..

[34] Vidal A., Claeys L., Mengelers M., Vanhoorne V., Vervaet C., Huybrechts B., De Saeger S., De Boevre M. (2018). Humans significantly Metabolize and Excrete the Mycotoxin Deoxynivalenol and its Modified Form Deoxynivalenol-3-Glucoside within 24 Hours. Sci. Rep..

[35] Edwards S.G. (2004). Influence of Agricultural Practices on Fusarium Infection of Cereals and Subsequent Contamination of Grain by Trichothecene Mycotoxins. Toxicol. Lett..

[36] Krupinsky J.M., Bailey K.L., McMullen M.P., Gossen B.D., Turkington T.K. (2002). Managing Plant Disease Risk in Diversified Cropping Systems. Agron. J..

[37] Jouany J.P. (2007). Methods for Preventing, Decontaminating and Minimizing the Toxicity of Mycotoxins in Feeds. Anim. Feed Sci. Technol..

[38] Blandino M., Reyneri A., Vanara F., Tamietti G., Pietri A. (2009). Influence of Agricultural Practices on Fusarium Infection, Fumonisin and Deoxynivalenol Contamination of Maize Kernels. World Mycotoxin J..

[39] Brodal G., Hofgaard I., Eriksen G., Bernhoft A., Sundheim L. (2016). Mycotoxins in Organically Versus Conventionally Produced Cereal Grains and some Other Crops in Temperate Regions. World Mycotoxin J..

[40] Edwards S.G. (2017). Impact of Agronomic and Climatic Factors on the Mycotoxin Content of Harvested Oats in the United Kingdom. Food Addit. Contam. Part A.

[41] Karlsson I., Mellqvist E., Persson P. (2022). Temporal and Spatial Dynamics of *Fusarium* Spp. and Mycotoxins in Swedish Cereals during 16 Years. Mycotoxin Res..

[42] (2022). Monitoring of Mycotoxins and Other Contaminants in UK Cereals Used in Malting, Milling and Animal Feed (2019–2022).

[43] Kolawole O., De Ruyck K., Greer B., Meneely J., Doohan F., Danaher M., Elliott C. (2021). Agronomic Factors Influencing the Scale of *Fusarium* Mycotoxin Contamination of Oats. J. Fungi.

[44] Schöneberg T., Jenny E., Wettstein F.E., Bucheli T.D., Mascher F., Bertossa M., Musa T., Seifert K., Gräfenhan T., Keller B. (2018). Occurrence of Fusarium Species and Mycotoxins in Swiss oats—Impact of Cropping Factors. Eur. J. Agron..

[45] McCormick S.P., Price N.P.J., Kurtzman C.P. (2012). Glucosylation and Other Biotransformations of T-2 Toxin by Yeasts of the Trichomonascus Clade. Appl. Environ. Microbiol..

[46] Wetterhorn K.M., Newmister S.A., Caniza R.K., Busman M., McCormick S.P., Berthiller F., Adam G., Rayment I. (2016). Crystal Structure of Os79 (Os04g0206600) from *Oryza sativa*: A UDP-Glucosyltransferase Involved in the Detoxification of Deoxynivalenol. Biochemistry.

[47] Yoshinari T., Sakuda S., Furihata K., Furusawa H., Ohnishi T., Sugita-Konish Y., Ishizaki N., Terajima J. (2014). Structural Determination of a Nivalenol Glucoside and Development of an Analytical Method for the Simultaneous Determination of Nivalenol and Deoxynivalenol, and their Glucosides, in Wheat. J. Agric. Food Chem..

[48] Sulyok M., Berthiller F., Krska R., Schuhmacher R. (2006). Development and Validation of a Liquid Chromatography/Tandem Mass Spectrometric Method for the Determination of 39 Mycotoxins in Wheat and Maize. Rapid Commun. Mass Spectrom..

[49] Varga E., Glauner T., Köppen R., Mayer K., Sulyok M., Schuhmacher R., Krska R., Berthiller F. (2012). Stable Isotope Dilution Assay for the Accurate Determination of Mycotoxins in Maize by UHPLC-MS/MS. Anal. Bioanal. Chem..

[50] Steiner D., Krska R., Malachová A., Taschl I., Sulyok M. (2020). Evaluation of Matrix Effects and Extraction Efficiencies of LC-MS/MS Methods as the Essential Part for Proper Validation of Multiclass Contaminants in Complex Feed. J. Agric. Food Chem..

