# Supplementary material for: Free and Modified Mycotoxins in Organic and Conventional Oats (Avena sativa L.) Grown in Scotland
Source: Toxins (Basel). 2023 Mar 28;15(4):247. doi: 10.3390/toxins15040247 (PMC10146303; doi:10.3390/toxins15040247)
Supplement: Supplementary file 1 [file toxins-15-00247-s001.zip › toxins-2282217-supplementary.pdf]

Oats for Safe and Healthy Nutrition Study – harvest 2019

FARM AGRONOMY QUESTIONNAIRE

Please complete the following questions where they are applicable to your growing system, for each sample and return with your oat sample.

NOTE: You should return your sample AND this completed questionnaire to Hamlyn's

According to the Project Privacy Policy, all samples will be anonymised before publication of any results and no individual data will be released to any party outwith the project partner organisations.

SECTION 1 (Completed by grower)

Farm details:

Farmer / Business name: ______________________________

Address: ______________________________

Post Code ______________________________

Tel number – landline ______________________________

Mobile: ______________________________

Email address ______________________________

1. Details of oat crop selected for survey:

1.1 Field size (ha): ______________________________

1.2 Name of field: ______________________________

1.3 Rotation:

2020 (planned)	
2018	
2017	
2016	
2015	

2. **2019 crop details:**

2.1 Variety: ____

2.2 Did you use Home Saved Seed? **Yes ☐ No ☐**

2.3 What seed dressings were applied?

Product	Date Applied	Rate

2.4 Date sown: ____

2.5 Seed rate/row spacing: ____________________________________

2.6 Details of seedbed preparation (complete what is applicable in this table):

	Yes	No	Date
**Ploughed**	☐	☐	
**Disced**	☐	☐	
**Rotavated**	☐	☐	
**Rolled**	☐	☐	
**Undersown with grass seed mix**	☐	☐	
**Other (please specify)**			

2.7 Do you know the soil analysis of the field?

If so, please complete the following:

**Date of analysis**	
**pH**	
**N**	
**P**	
**K**	
**Micronutrient A (please state)**	
**Micronutrient B (please state)**	
**Micronutrient C (please state)**	

3. Nutrients

3.1 Did you apply any naturally-composted materials (eg Farm yard Manure, Digestate etc)?

Yes ☐ **No** ☐ If Yes, please provide the following:

Type of composted material	Date Applied	Application Rate	Nutrient Analysis of product (if available)	Moisture content of product at time of application (if available; if not, then what was your visual assessment)
**Well composted FYM (>6 months composted**				
**Fresh FYM (<6months old)**				
**Digestate**				
**Green Waste Compost**				
**Other (please state)**				

3.2 What Inorganic fertiliser was applied to 2019 crop, if applicable?

Nutrient	Date Applied	Application Rate	Nutrient Analysis of Product (if available)
**N**			
**P**			
**K**			
**Lime (which product?)**			
**Other (please state)**			
**Other (please state)**			

3.3 If applicable, what crop protection products (herbicides, fungicides, growth regulators, desiccants, foliar nutrients etc) were applied to the growing crop:

Product	Rate	Date Applied	Reason

4. Weather data:

**Rainfall at flowering (GS59-69)**	**More than 80mm**
	**40-80mm**
	**10-40mm**
	**Less than 10mm**
**Rainfall pre-harvest (GS87- harvest)**	**More than 120mm**
	**80-120mm**
	**40-80mm**
	**20-40mm**
	**Less than 10mm**

5. Harvest details:

5.1 Date of harvest: ____

5.2 Grain Moisture content at time of harvesting: ________________________________

Date of drying	Drying method ***no other post-harvest processing to be conducted before sampling***	Final moisture content

6. **Sampling – *Use method described at bottom of page to obtain a representative sample from selected crop:**

6.1 Confirm that the harvested selected crop has not been bulked with any other oats at time of sampling: **Yes** ☐ **No** ☐

6.2 Date sample taken – use your unique sample bag provided: ________________________

6.3 Sample taken by: : ________________________

***How to obtain a representative sample – follow the instructions:**

This is achieved by taking 20 mug-sized individual samples from different positions/depths in the bulk (incremental samples) to form an aggregate sample in a bucket– a large sample comprising all smaller samples. This will be much larger than the 'final' sample retained as representative sample. The aggregate sample should be thoroughly mixed and finally a representative sample of approximately 1kg taken from the bucket and stored in the sample bag.

6.4 Date sample sent to lab: ________________________

SECTION 2 – PROJECT TEAM

2.1 To be completed by Primary Collector Hamlyn's

2.1.1 Date Sample Received ____

2.1.2 Reference allocated to sample: ____

2.1.3 Date sent for Sample Preparation: _____________________________________

2.1.3 Staff member undertaking sample preparation _____________________________

2.1.4 Details and Date of sample preparation: __________________________________

2.2 Aberdeen University

2.2.1 Date sent to Aberdeen University**:** ____

2.2.2 Details of transportation method: ____

2.2.3 Date received at Aberdeen University: ____________________________________

2.2.4 Staff member analyzing sample: ____

2.2.5. Reference allocated to sample (this should be the same as 2.1.2)

____

2.2.6 Date sample database updated: ____
